# Iron Status and Supplementation during Tuberculosis

**DOI:** 10.3390/microorganisms11030785

**Published:** 2023-03-18

**Authors:** Arista Nienaber, Mary A. Uyoga, Robin C. Dolman-Macleod, Linda Malan

**Affiliations:** Centre of Excellence for Nutrition, North-West University, Potchefstroom 2531, South Africa

**Keywords:** anaemia of infection, iron, iron deficiency anaemia, tuberculosis

## Abstract

Tuberculosis (TB) is characterised by chronic non-resolving inflammation. The effects of the host immune and inflammatory response to reduce iron acquisition by the bacteria, together with other contributing factors, predispose TB patients to anaemia of infection and iron deficiency anaemia (IDA). The presence of anaemia in TB patients has been linked to poor clinical outcomes. However, due to the reliance of the bacteria on iron, the management of anaemia in TB is complicated, and anaemia of infection is likely to resolve with correct TB drug treatment. On the other hand, IDA may require iron supplementation. This review aims to describe iron metabolism in TB and how this contributes to the development of iron deficiency and anaemia. Additionally, we summarise the evidence on the association between iron status and clinical outcomes as well as the available preclinical and clinical trials on iron supplementation in TB.

## 1. Introduction

Tuberculosis (TB) is still a leading cause of morbidity and mortality in the world, with increasing drug-resistant cases. The prevalence of bacteriologically confirmed pulmonary TB cases ranges from 24 to 62% in African countries and 33 to 68% in Asian countries according to a National WHO survey in 24 countries during 2007–2016 [1,2,3]. 

Tuberculosis patients are burdened with excessive non-resolving inflammation with resultant debilitating consequences, including lung tissue necrosis and cavitation [4,5]. Furthermore, anaemia, also resulting from inflammation, is a common complication in TB patients. Normocytic normochromic anaemia has been reported to be present in up to 71.7% of TB patients [6,7,8,9,10,11,12,13]. Additionally, depending on the population and the relevant contributing factors, 1-53% of TB patients suffer from iron deficiency anaemia (IDA) [8,9,14]. Populations at risk for TB are frequently from a low-socio-economic background, where undernutrition may lead to micronutrient deficiencies, including iron deficiency (ID) [15,16]. Furthermore, intestinal helminth infection before TB infection and co-infection of TB patients with helminths may cause IDA [17,18]. Due to the importance of iron in immunity, ID and anaemia in TB patients have been associated with slower sputum conversion rates, higher mortality rates and the reoccurrence of TB [3,7,8,9]. However, the relationship between iron availability, the TB bacterium and the host immune response is complex; therefore, it is difficult to identify the need and benefit of iron supplementation [19,20]. This review aimed to summarise the literature available on ID and anaemia in TB and the possible role of iron supplementation in addressing these complications. 

## 2. Iron Acquisition and Host Immune Response in Tuberculosis

**Figure 1:**
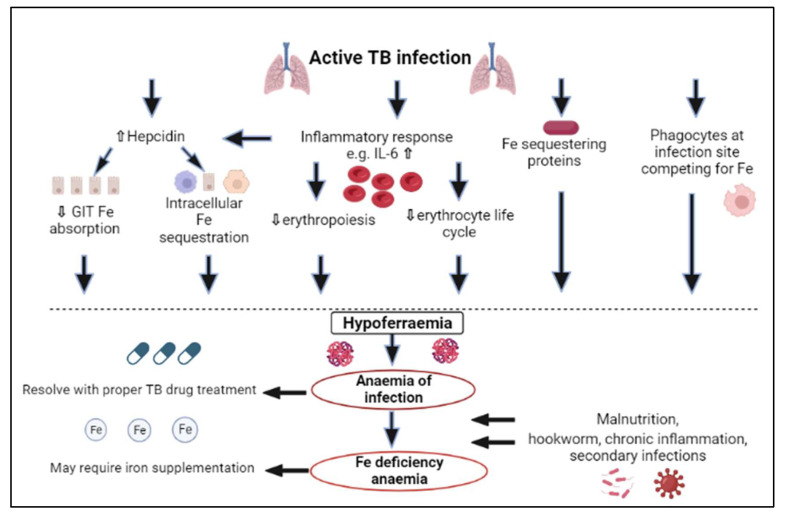
The pathophysiology of the development of iron deficiency and anaemia in tuberculosis. IL: interleukin, Fe: iron, GIT: gastrointestinal, TB: tuberculosis.

Iron serves as a co-factor for the metabolic enzymes of mycobacteria such as aconitase and succinate dehydrogenase and is therefore essential for their subsistence and replication [21,22,23]. Numerous microbes depend on iron obtained from the host, which is also true for *Mycobacterium tuberculosis* (*Mtb*), which uses the host’s iron for growth and virulence [20]. For this reason, the role of the innate immune response is to lower iron accessibility for *Mtb*, as illustrated in Figure 1 [19,24,25]. 

The first way in which iron is restricted for the bacteria is through iron-sequestering proteins present in mucosal fluids. Lactoferrin is such an example and, like transferrin, binds with two ferric atoms. Iron is not released from lactoferrin even if the environment is very acidic, and consequently, it provides part of the initial barrier against iron availability for bacteria [26]. An additional iron-sequestering protein is lipocalin-2, which acts by isolating iron-binding bacterial siderophores and using catechol metabolites as iron chelators [27,28]. In TB, it has been established that extracellular iron-sequestering molecules, for example, transferrin, which transports ferric ions; haptoglobin, which is responsible for binding haemoglobin; and hemopexin, which sequesters free haem iron, gather in the necrotic centres of caseous granuloma. This is accompanied by iron-restricting antimicrobic proteins, for example, lactoferrin and lipocalin, which isolate siderophores and impede *Mtb* growth in granulomas [29]. All these iron-restricting proteins that are in granulomas reduce the availability of iron for *Mtb*. Additionally, at infection sites, phagocytes are also recruited to compete with bacteria for iron [24] (Figure 1). 

Furthermore, during the acute-phase response, there are changes in plasma concentrations of proteins implicated in iron metabolism, such as transferrin, hepcidin and ferritin [24,30]. Following host *Mtb* recognition, a proinflammatory response is stimulated, which triggers liver hepcidin to be upregulated by the production of proinflammatory cytokines, predominantly interleukin (IL)-6, and additionally IL-1, IL-22 and interferon-gamma (IFN-α) [22,31,32,33,34,35,36]. Other factors related to infection, for example, the hydrogen peroxide that is produced by neutrophils, also influence the expression of hepcidin [37]. Elevated concentrations of hepcidin cause the sequestration of iron in macrophages, impaired gastrointestinal iron absorption and decreased release of stored iron. Hepcidin causes the endocytosis and proteolysis of ferroportin, together with reduced expression of divalent metal transporter-1, thereby reducing iron gastrointestinal absorption and enhancing iron sequestration intracellularly in macrophages, hepatocytes and enterocytes as cytoplasmic ferritin [33,38]. This is acknowledged as the primary cause of hypoferraemia during inflammation and infection (Figure 1) [39,40].

Additionally, there are other ways than the upregulation of hepcidin in which inflammatory cytokines play a role in the development of hypoferraemia (Figure 1). For example, IFN-γ mediates myeloid cell production at the expense of erythropoiesis and activates macrophages, leading to a shorter erythroid life cycle due to phagocytosis, whilst tumour necrosis factor-alpha (TNF-α) impedes erythroid proliferation. Cytokines such as TNF-α, which acts on duodenal cells to lower iron absorption independently of hepcidin, can further directly reduce iron absorption. IL-1, IL-6, IL-10 and TNF-α also act on macrophages by transferrin-receptor-mediated endocytosis, divalent metal transporter-1 and lactoferrin and lipocalin-2, thereby facilitating iron restriction [39,40,41,42]. 

In essence, in TB there is a change from iron that is available (transferrin bound) to iron that is stored (ferritin) [19,25,33]. This, however, avails the iron for intracellular microbes, such as *Mtb*. *Mtb* has restricted access to the host iron stores in the phagosome of host macrophages, but evidence shows that *Mtb* secretes siderophores that mediate iron diffusion through phagosomal membranes, thereby offering contact with the abundantly available iron in the macrophage cytosol [43]. Siderophores are molecules that can bind iron better than iron storage proteins and acquire iron from transferrin, lactoferrin and ferritin [43]. In TB, lipophilic, cell-bound mycobactins and soluble carboxymycobactins that are secreted into the extracellular space are such siderophores [43,44,45]. Additionally, extracellular *Mtb* attains iron from circulating transferrin, ferritin and lactoferrin. There are also ways in which host haem iron is obtained by *Mtb* as it expresses and secretes haemolytic enzymes that induce haemolysis of red blood cells (RBCs) to release haemoglobin and, therefore, in advanced stages of TB infection, high lung haem iron levels have been detected [43,46].

## 3. Iron Status in Tuberculosis

The mechanisms which are meant to lower the availability of iron for pathogens [46] also impact erythropoiesis by resulting in reduced iron absorption and serum iron availability (hypoferraemia) [21,22,47]. Three pathways have been suggested to contribute to anaemia of infection. These include hepcidin-mediated iron sequestration, the inflammatory inhibition of erythropoiesis and a shorter life cycle of erythrocytes [41]. The low amounts of iron transported by transferrin in the circulation result from the large portion of bodily iron constricted to the macrophages of the reticuloendothelial system. Iron production for other tissues, such as, the central nervous system and bone marrow precursors for leukocyte production, is preserved by inhibiting erythropoiesis [40,48]. In TB, ongoing inflammation causes hepcidin to be upregulated, which restricts iron availability for erythropoiesis and, in combination with reduced dietary and supplemental iron absorption, finally results in normocytic normochromic anaemia [33,40]. The availability of erythroid precursors is lowered by hypoferraemia, and the higher production of leukocytes further decreases the body’s oxygen-carrying capacity [22,33]. This anaemia is known as anaemia of infection, inflammation or chronic disease and, except for during critical illness, may take several weeks to develop. This is due to the long life cycle of erythrocytes in adults (100 to 120 days) [40]. 

Over a prolonged serious illness, patients could develop the more detrimental condition of microcytic hypochromic RBCs [33]. Anaemia of infection and IDA additionally regularly co-exist, particularly in developing countries [49]. Other factors may contribute to the development of IDA in TB. These may include the following [10,19,50,51,52]:

- Malnutrition because of insufficient dietary intake and a deterioration in appetite;
- Malabsorption because of hookworm infection, inflammation or other secondary infections;
- Haemoptysis resulting in blood loss via sputum.

Supporting this, Chhabra et al. reported that the degree of malnutrition is associated with the presence of anaemia in TB patients [47]. 

Anaemia of infection and IDA are common complications in TB, being reported to be present in 30 to 94% of TB patients [6,8,10,13,14,19,51,52,53,54]. These anaemias can either be TB-related or from alternative causes. Normocytic normochromic anaemia (anaemia of infection) is the main anaemia present in TB patients [6,7,8,9,10,11,12,13,53]. However, Isanaka et al. found a high IDA burden of 53% in the TB patients included in their study in Dar es Salaam, Tanzania [14]. Another study reported that of 198 Iranian smear-positive pulmonary TB patients, 45.9% were anaemic at the end of the second month of TB drug treatment, and of these patients, 79.1% suffered from IDA [55]. Anaemia of infection improves with lowered hepcidin concentrations and iron availability in the circulation when there is effective TB treatment and infection resolution [56]. However, IDA may persist, and iron-based interventions for these patients may be needed [8,11,14].

## 4. Biomarkers to Identify Anaemia of Infection and Iron Deficiency Anaemia in Tuberculosis patients

The fact that anaemia of infection and IDA are both characterised by low iron levels (hypoferraemia) makes distinguishing between the two challenging. However, there are differences in that in IDA, lower ferritin concentrations are observed in comparison with higher hepcidin and ferritin concentrations in anaemia of infection [40]. 

When investigating the relationship of TB with iron status biomarkers, Mishra et al. found that compared with healthy individuals, pulmonary TB patients had higher ferritin, total iron-binding capacity and C-reactive protein (CRP) concentrations [19]. This has been confirmed by past and more current research that reported elevated ferritin levels in TB patients [8,12]. Kurthkoti et al. measured ferritin concentrations on a cellular level and reported high concentrations in cavitary granulomas’ cellular regions [29]. Ferritin concentrations were also found to shift with TB drug treatment. Therefore, ferritin is instead defined as an acute-phase protein in TB patients and is not an accurate indicator of iron stores under these circumstances as it is influenced by the resolution of infection and inflammation [19]. Ferritin levels are thought to increase in TB due to two mechanisms: firstly, the fact that monocytes and macrophages produce ferritin and monocytosis is a consequence of TB infection, and secondly, ferritin acts as an acute-phase reactant that is therefore closely associated with CRP (inflammation) [19]. 

Low iron, transferrin, transferrin saturation (TSAT) and haemoglobin levels have been found in TB patients [8,19]. Due to the fact that transferrin is considered a negative acute-phase protein, the reduction in transferrin concentrations concurs with what is anticipated in infections [19]. Moreover, as discussed above, the shift of circulating iron to being stored (i.e., as ferritin) is also why reduced transferrin levels are evident in TB patients. Lastly, transferrin is influenced by inflammation as well as being a nutritional status marker affected by the intake of protein and iron [19]. Conversely, as an indication of higher iron demand and erythropoietic stimulus, elevated transferrin receptor (TfR) levels can be expected in inflammatory situations [57]. TfR levels are, therefore, a poor marker of IDA also affected by inflammation. Kurthkoti et al. found elevated TfR in solid cellular granulomas [29], which was supported by a study in Tanzanian TB patients that found higher circulatory TfR levels [8]. Furthermore, when comparing TB patients with and without anaemia, no significant difference could be found in their TfR levels [58]. 

The fact that lower hepcidin concentrations are anticipated in IDA and higher concentrations in anaemia of infection means that hepcidin’s upregulation is a potentially helpful way to differentiate amongst these two types of anaemia in TB patients [59]. Higher hepcidin levels have repeatedly been reported in TB patients [8,60,61]. Table 1 shows the differences in the shifts in biomarkers seen in patients with anaemia of infection compared with IDA.

## 5. Clinical Outcomes Associated with Iron Biomarkers in Tuberculosis

Hypoferraemia and anaemia have consequences for TB patients [19,24,44,48,49]. Firstly, the sequestration of iron upon TB infection benefits *Mtb* pathogens that inhabit macrophages [20]. In addition, iron plays a vital role in both innate and adaptive immune function [21,50,51]. The unavailability of iron inhibits cell defence systems and macrophage activity by causing a change from Th1 towards Th2 responses elicited by reduced IL-1 and IL-6 concentrations. This results in an inhibited ability of macrophages to kill the mycobacteria and block nitrogen-oxide-dependent activity [14,52,53,54,55]. ID further compromises immune function and response by reducing T-cell numbers and the inaccessibility of iron to be part of the structure or to activate enzymes required for the immune response [50,56,57,58,59]. Apart from affecting the immune response, ID also has other unfavourable consequences because iron is required for physiological processes such as erythropoiesis [19]. Therefore, ID and anaemia can precipitate various symptoms, including weakness and impaired motor activity [19]. 

Due to the effects of ID on immune function and other physiological functions and the intracellular shift of iron favouring bacterial growth, ID and anaemia are also risk factors for poor TB outcomes [24]. The relationship between iron status and TB is complex because both ID and overload may increase an individual’s susceptibility to and progression of TB infection [19,24,51,60,61]. Chu et al. showed that individuals with IDA have a significantly higher TB incidence compared with non-IDA controls [62]. A Tanzanian trial in TB patients by Isanaka et al. first demonstrated significant associations between ID and anaemia and negative clinical outcomes and recurrence of disease [14]. Here, anaemia with and without ID was related to a two- to threefold independent rise in the risk for mortality [14]. Additionally, anaemia with no ID was also related to TB recurrence [14]. These findings are supported by other studies in TB patients, indicating an association between anaemia and delayed sputum conversion and higher mortality rates [61,63]. However, Metanat et al. could not identify a significant difference in sputum conversion rates between IDA and non-IDA pulmonary TB patients after the intensive TB drug treatment phase [64]. 

Several markers of iron status have been shown to be associated with the advancement of TB disease and clinical outcomes. Plasma or serum ferritin levels correlate positively with disease severity, sputum positivity and higher mortality rates during active TB [19,65]. However, reduced levels of plasma ferritin at infection, before active TB, predicted a greater risk of failed treatment and TB recurrence in HIV-positive patients [65]. In addition, a trial in South Africa reported a strong correlation between hepcidin concentrations and mycobacterial burden and that hepcidin predicted mortality in TB patients [66]. Similarly, a study in Tanzania found a positive correlation between hepcidin and the severity of TB symptoms [8]. 

Apart from ferritin and hepcidin, low transferrin and haemoglobin and elevated ferritin were consistent with a higher TB incidence and susceptibility in HIV patients [67]. Similarly, other studies have also shown a strong correlation between low transferrin levels and TB disease severity [19,68,69]. Finally, an older study found a positive correlation between haemoglobin levels and faster rates of sputum conversion [12]. Evidently, in TB patients, ID and anaemia may lead to unfavourable consequences that can be attenuated by improving the individual’s iron status, thus improving outcomes [65]. 

## 6. Preclinical Studies of Iron Supplementation and Absorption in Tuberculosis

Treating ID in TB is not straightforward, as providing iron to guarantee competent immune functioning results in iron that is available for pathogens [20,70,71]. Currently, no clear guidelines exist for treating ID during infection [24,51]. In addition, due to the upregulated inflammatory response in TB patients, iron supplementation may fail to correct anaemia of infection. Treatment strategies aimed either at the infection or inflammation that may resolve anaemia of infection and TB symptoms, e.g., anti-TNF therapy, have been suggested [10,72]. However, some studies have also emphasised that iron supplementation may be beneficial to improve iron status in some TB patients [8,14]. Intravenous iron therapy has been suggested for patients suffering from both anaemia of inflammation and IDA [39]. Nevertheless, research on iron supplementation in TB patients and animal models is limited. Table 2 summarizes the available preclinical trials investigating iron availability and administration in TB. 

Serafin-Lopez et al. found that in TB-infected murine J774 macrophages, iron favoured *Mtb* intracellular growth due to the reduced production of TNF-α, which is necessary for *Mtb* restriction [54]. Similarly, a study using human mononuclear phagocytes showed that iron repressed the release of TNF-α while attenuating the reaction of monocytes to TNF-α. The researchers reasoned that this is a requirement for the differentiation of monocytes, and a necessary step in limiting the cell-to-cell spread of *Mtb* [73]. 

Iron supplementation outcomes in animal experiments have been inconsistent (Table 2). When *Mtb*-infected BALB/C mice were intraperitoneally injected with 50 mg/kg polymaltose ferric hydroxide thrice daily for two weeks before infection, this resulted in mice with a high iron load having greater bacterial loads in their lungs and spleens [74]. Similarly, Schaible et al. found that in β-2-microglobulin knockout mice, iron overload worsened TB [75]. Conversely, recent animal trials found otherwise. In one study, rabbits with latent TB infection were injected with 25 mg of iron thrice every week for 2 months. This study found no effects on haemoglobin or haematocrit. However, in the iron-supplemented groups, total plasma iron-binding capacity and percentage TSAT were reduced and iron concentrations in the lungs were elevated [76]. Supplementation with iron did not affect either the pathology of the disease or the bacterial loads but downregulated systemic immune response gene expression such as *IL1B*, *IL10*, *TNFA* and *IFNG* while upregulating lung and plasma *IL6* expression [76]. Additionally, Agoro and colleagues fed *M.Bovis* BCG-infected C57BL/6 mice with a diet consisting of either 2500 (supplemented) or 280 mg of iron carbonyl per kg diet [77]. This study found that the lungs of iron-supplemented mice had lower levels of proinflammatory cytokines including IFN-γ, IL-1β, IL-12p40 and TNF-α. Additionally, supplementation enhanced the recruitment of immune cells, resulting in higher CD8^+^ T cell counts [77]. In another study, oral iron supplementation in *Mtb*-infected C3HeB/FeJ mice commencing one week post-infection for three weeks had a lowering effect on soluble TfR, ferritin and hepcidin levels. Iron also lowered concentrations of IL-1α and IL-1β in the lungs and IL-1β, TNF-α and IL-6 in plasma [78]. The reason for the discrepancies between the results found in preclinical studies are possibly due to differences in the TB models used or methods and dosages of TB infection, as well as the timing, dosage and route of iron administration.

**Table 2:** Preclinical studies investigating the relationship between iron and TB.

Reference	TB Model	Iron intervention	Outcome
Byrd et al. [73]	Human monocytes infected with *Mtb* Erdman strain (ATCC 35801) and cultured in medium containing TNF-α, IFN-γ and calcitriol.	Ferric ammonium citrate, Fe-saturated transferrin, Fe-saturated lactoferrin.	Dose-dependent Fe restriction of growth of *Mtb* in monocytes that had been primed with TNF-α. Production of TNF-α by infected monocytes was inhibited by Fe.
Lounis et al. [74]	Balb/C mice infected intravenously with 7.2 × 10^3^ H37Rv *Mtb* strain.	50 mg/kg polymaltose ferric hydroxyde intraperitoneally, 3 times a week for 2 weeks before infection.	No significant differences in body weights between the Fe-loaded and control mice on day 42 after infection. Spleen weights were significantly higher in Fe-loaded mice at day 42 post-infection. *Mtb* CFU counts significantly higher in spleens and lungs of the Fe-loaded mice compared to controls.
Schaible et al. [75]	In vivo wild-type B6, β-2-microglobulin knockout and MHC-I knockout mice infected with 3–5 or 15–200 *Mtb* per lung. Some mice received 25 mg/mL ferric citrate in drinking water for duration of experiment to overload them with Fe.	Treated twice every week with intranasal 1 mg/mouse bovine lactoferrin or recombinant lactoferrin or intraperitoneally with deferoxamine in PBS or PBS alone. 25 mg/mL Fe^3+^Ci	Treatment with Fe^3+^Ci resulted in Fe overload and 10 times higher burden of lung *Mtb*. Treatment with lactoferrin ↓ bacterial load in β2m knockout but not B6 mice. Treatment with deferoxamine depleted Fe and ↓ bacterial load in both β2m knockout and B6 mice.
	In vitro *Mtb* (Erdman) and *M.bovis* cultured in medium.	1 mg/mL Fe^3+^Ci; or 1 mg/mL bovine lactoferrin; or 0.5 mg/mL deferroxamine; or deferroxamine and Fe^3+^Ci.	Excess Fe ↑ bacterial growth; deferoxamine chelated free Fe, resulting in ↓ growth of *Mtb.*
	Macrophages derived from bone marrow cells of B6 or β2m knockout mice were infected with *Mtb* and cultured with or without 0.5 mg/mL lactoferrin.	0.5 mg/mL lactoferrin or 0.1 mg/mL rat anti-TfR antibody for 3 days.	Lactoferrin bound extracellular Fe, whereas anti-TfR antibody inhibited cellular Fe import, thus inhibiting growth of *Mtb* in infected macrophages.
Serafin-Lopez et al. [54]	Murine macrophage-like cell line J774A.1 infected with *Mtb* H37Rv bacilli. Intracellular and extracellular growth of *Mtb* assessed.	Incubation of cell cultures with 5, 25 and 50 µM of ferric chloride for 0, 48 and 72 h.	Dose-dependent extracellular bacterial growth was observed after 48 h and 72 h but intracellular growth only at 72 h with Fe. Production of TNF-α was lower at 72 h compared with 48 h post-infection in macrophages with Fe.
Agoro et al. [77]	Male C57BL/6 mice infected intravenously with 2 × 10^6^ *M. Bovis* BCG.	Mice on (1) Fe-rich diet (2500 mg Fe carbonyl/ kg food or (2) standard diet (280 mg Fe carbonyl/ kg food) for 4 weeks before infection plus duration of infection.	In vivo analysis Moderate Fe: ↓ proinflammatory cytokine levels, ↓ neutrophil recruitment, ↑ T-cell recruitment in granulomas and ↓ bacterial load. ↑ Bacterial clearance in liver correlated with upregulation of the gene encoding hepcidin and sequestration of Fe in tissues. In cultured macrophages Fe ↑ ROS and ↓ uptake and intracellular growth of *M.Bovis* BCG.
Kolloli et al. [76]	Pathogen-free white rabbits (*Oryctolagus cuniculus*) infected with *Mtb* CDC1551 via the aerosol route.	25 mg Fe-dextran III or placebo (0.5 mL sterile dextran in water) intra-muscularly, 3 days a week. (1) For acute-phase results starting day 1 post-infection to 8 weeks and (2) for pre-established infection starting at 8 until 16 weeks.	No causal role for Fe in bacterial burden and tissue pathology, i.e., the reactivation of latent TB. Association between Fe supplementation and changes in host gene expression of Fe homeostasis and host immunity.
Nienaber et al. [78]	Male C3HeB/FeJ mice infected with 2.4 × 10^7^ *Mtb* H37Rv via aerosol route.	AIN-93G control or AIN-93G diet supplemented with Fe (130 ppm Fe) from one-week post-infection for three weeks.	Fe lowered soluble transferrin receptor, ferritin and hepcidin, lung IL-1α, IL-1β, plasma IL-1, IL-6 and TNF-α. Fe did not affect lung bacterial loads. Fe ↑ T-cell, CD4+ T-cell, CD8+ T-cell, interstitial macrophage, alveolar macrophage, CD103 DC and CD11b DC counts in lungs and percentages of total lung cells in neutrophils, interstitial macrophages, monocyte-derived DCs, T cells, CD4+ T cells, CD8+ T cells, natural killer cells and CD11b DCs.

AGP: α -1-acid glycoprotein, *M.bovis* BCG: *M.bovis* bacillus Calmette–Guérin, CRP: C-reactive protein, DCs: dendritic cells, Fe: iron, Fe^3+^Ci: iron citrate Hb: haemoglobin, IL: interleukin, *Mtb*: *mycobacterium tuberculosis,* ROS: reactive oxygen species, sTfR: soluble transferrin receptor, TSAT: transferrin saturation, TNF-α: tumour necrosis factor-alpha, PBS: phosphate-buffered saline, IFN-γ: interferon-gamma, ↑: higher, ↓: lower.

## 7. Clinical Trials on Iron Supplementation and Absorption in Tuberculosis

Currently, there are few clinical trials investigating iron supplementation in TB patients. Murray et al. examined iron supplementation in two iron-deficient TB patients. The authors concluded that disease activity was increased with iron supplementation and that infection was most probable with repleted iron stores [70]. This was supported by another study that showed that dietary iron intake together with elevated macrophage iron stores was positively correlated with active TB and mortality risk (1.3-fold increased hazard ratio of death) [19,53,60]. Similarly, intravenous iron administration favoured the activation of TB in a published case study [79].

Countering the above, Devi et al. found that supplementation of pulmonary TB patients with 75 mg of ferrous fumarate twice daily elevated circulating haemoglobin, total mean cell volume, erythrocyte count and packed cell count a month after therapy commencement, but this did not continue after the second month of treatment, irrespective of whether supplementation was sustained [6] (Table 3). It was argued that the initial quicker improvement in biochemical markers decelerated as additional improvements were affected to a greater extent by stabilising inflammation and not by the supplementation of iron per se. Supporting this notion, total iron-binding capacity was found not to be dependent on supplementation and more reliant on the resolution of inflammation [6]. The authors also reported that neither body mass index nor radiological improvement (chest lesion severity) was influenced by supplementation with iron. They hypothesised that there is a regulatory mechanism involved in TB infection to acquire host iron, regardless of the iron status of the host [6].

Cercamondi et al. recently investigated iron metabolism in TB patients undergoing treatment [80] (Table 3). Their study found that iron absorption from 6 mg of labelled iron as ferrous sulphate was negligible before treatment (0.8%) but increased 10- and 20-fold to 8% and 15.2% after intensive TB drug treatment (2 months) and completion of treatment (a further 4 months), respectively [80]. Furthermore, the high burden of anaemia observed at baseline, possibly due to inflammation, resolved and haemoglobin steadily increased until the end of treatment [80]. Similarly, serum iron concentrations and transferrin saturation increased significantly with treatment administration compared to baseline, consistent with the initially sharp and then gradual decline in hepcidin and IL-6 concentrations that was observed during the treatment period [80]. The study also showed that erythroferrone, the hormone that usually stimulates the formation of erythrocytes by suppressing hepcidin, was only able to do so once the inflammation started resolving [80]. Based on their findings, the authors concluded that iron supplementation in TB patients should be initiated only upon completion of TB drug treatment when absorption will be most efficient, and only for patients that remain anaemic [80].

Taken together, the results of preclinical and clinical studies suggest that restricting the host’s persistent inflammation induced by TB may make a significant contribution to repleting iron stores and addressing anaemia. However, iron supplementation may not worsen disease progression and may have anti-inflammatory effects. In addition, in a review, Agoro and Mura proposed that the effect of iron supplementation in mycobacterial infections is biphasic [20]. Moderate iron supplementation within the “iron benefit window” may be beneficial for the host response to reduce the bacterial load and inflammation. However, iron supplementation exceeding the limit of the “iron benefit window” can have unfavourable effects, promoting bacterial growth and virulence, with poor outcomes [20].

## 8. Conclusions

*Mtb* bacilli require iron for growth and virulence. For this reason, there are various mechanisms by which the host response aims to reduce iron availability. The heightened inflammatory response with hepcidin, iron-sequestering proteins and phagocytes competing for iron at infection sites and lower erythropoiesis all contribute to lower iron availability for *Mtb*. However, this also causes hypoferraemia in the host, which eventually precipitates in anaemia of infection. Other contributing factors, such as secondary infections and poor nutrition, may further predispose a TB patient to IDA. It is difficult to distinguish between anaemia of infection and IDA, which may present with similar iron status marker derangements, as many of these biomarkers are influenced by inflammation. Nevertheless, proper diagnosis of a true IDA is required for the effective treatment of patients.

Anaemia of infection and IDA have been linked to poor clinical outcomes. However, there are no clear guidelines on whether TB patients presenting with ID and anaemia require iron supplementation. It is generally accepted that anaemia of infection will resolve with standard TB drug treatment. IDA, especially when persisting after TB treatment and cure, may require some further consideration of iron supplementation.

Preclinical studies on iron supplementation in TB have yielded mixed results, and only two clinical trials could be identified. Most recent studies have shown that moderate, timely iron supplementation during TB infection does not worsen bacterial load and disease progression and may have an inflammation-lowering effect. However, more clinical trials are required to determine the exact dosage and timing of iron administration as well as the conditions under which supplementation is warranted.

## Figures and Tables

**Table 1:** Biomarker changes anticipated in anaemia of infection and iron deficiency anaemia.

Biomarker	Levels in Iron Deficiency Anaemia	Levels in Anaemia of Infection
Red blood cells	Low	Low
White blood cells	Low to normal	Normal to high
Serum iron	Decreased	Decreased
Serum transferrin	High	Low
Transferrin saturation	Low	Low
Serum transferrin receptor	High	Normal to high
Hepcidin	Decreased	Increased
Haemoglobin	Decreased	Decreased to normal
Ferritin	Low	High
Mean corpuscular volume	Low	Normal
Mean corpuscular haemoglobin	Low	Normal

**Table 3:** Clinical studies investigating iron supplementation in TB patients.

Reference	Participants	Iron intervention	Outcome
Devi et al. [6]	n = 117 male PTB patients 15–60 years old and Hb of 80–110 g/L n = 50 healthy male controls matched for age and socio-economic status.	1 capsule twice/ day of (1) placebo containing 75 mg of sucrose; (2) ferrous fumarate containing 75 mg of elemental Fe; (3) ferrous fumarate containing 75 mg of elemental Fe with other haematinics for 2 months while hospitalised.	↑ Hb, MCV and PCV at 1 month in both Fe-supplemented groups. This difference disappeared at 2 and 6 months. Serum Fe and Fe saturation of transferrin was ↑ in both Fe-supplemented groups for up to 2 months. Radiological and clinical improvements similar in all three groups.
Cercamondi et al. [80]	n = 18 mostly anaemic men and women aged 16–45 years old with a positive sputum smear confirmed by gene expert.	Test meal with 6mg of ^57^Fe iron as ferrous sulphate administered at baseline then 8 and 24 weeks post-treatment. At baseline intravenous infusion with ^54^Fe or ^58^Fe as Fe^3+^Ci, after a meal.	Significant ↓ in inflammation markers: AGP, CRP and IL-6, and 70% ↓ in hepcidin at 2 weeks. Haemoglobin ↑ and anaemia prevalence ↓ from 89% to 22%. TSAT ↑ and ferritin ↓ significantly but sTfR did not change from baseline until treatment completion. Negative correlation between hepcidin and serum iron, indicating Fe sequestration during infection, weakened upon treatment. At baseline, inflammation resulted in Fe sequestration and inhibited absorption and erythropoiesis. With treatment, hepcidin was suppressed and erythropoiesis upregulated for recovery of Hb. Fractional Fe absorption increased 10- and 20-fold as infection resolved.

Fe: iron, Fe^3+^Ci: iron citrate, Hb: haemoglobin, IL: interleukin, MCV: mean cell volume, PCV: packed cell volume, PTB: pulmonary TB, sTfR: soluble transferrin receptor, TB: tuberculosis, TSAT: transferrin saturation, ↑: higher, ↓: lower.

## Data Availability

Not applicable.
